# Large bowel mucosal neoplasia in the original specimen may increase the risk of ileal pouch neoplasia in patients following restorative proctocolectomy for ulcerative colitis

**DOI:** 10.1007/s00384-015-2271-1

**Published:** 2015-05-29

**Authors:** Adam Bobkiewicz, Lukasz Krokowicz, Jacek Paszkowski, Adam Studniarek, Krzysztof Szmyt, Jan Majewski, Jaroslaw Walkowiak, Przemyslaw Majewski, Michal Drews, Tomasz Banasiewicz

**Affiliations:** Department of General, Endocrinological and Gastroenterological Oncological Surgery, Poznań University of Medical Sciences, Przybyszewskiego 49, 60-355 Poznan, Poland; Department of Clinical Pathomorphology, Poznań University of Medical Sciences, Przybyszewskiego 49, 60-355 Poznan, Poland; Department of Pediatric Gastroenterology and Metabolism, Poznań University of Medical Sciences, Szpitalna 27/33, 60-572 Poznan, Poland

**Keywords:** Restorative proctocolectomy, Ileal pouch, Ulcerative colitis, Neoplasia

## Abstract

**Purpose:**

Restorative proctocolectomy is a current gold standard procedure for patients who require a colectomy for ulcerative colitis. The incidence of ileal pouch neoplasia is low. The aims of this study were to assess the prevalence of neoplasia in ileal pouch and investigate the risk factors for ileal pouch neoplasia.

**Methods:**

A total of 276 patients who underwent restorative proctocolectomy for ulcerative colitis between 1984 and 2009 were analyzed. Results of histological examinations of both original specimen and biopsies from the J-pouch taken during routine pouch endoscopy were evaluated. Patients’ records were analyzed for ulcerative colitis duration, the time from pouch creation to pouch neoplasia, presence of pouchitis, as well as the concurrent primary sclerosing cholangitis.

**Results:**

Analyzing the original specimen of large bowel, fifty-six lesions of low-grade dysplasia, twenty-five high-grade dysplasia, and five adenocarcinoma were revealed. All patients with dysplasia (*n* = 8) or adenocarcinoma (*n* = 1) of the J-pouch were positive for dysplasia in the original specimen. Duration of ulcerative colitis before surgery and duration time following restorative proctocolectomy were found as risk factors for J-pouch neoplasia with a significant difference (*p* = 0.01 and *p* = 0.0003, respectively). Patients with pouch neoplasia developed significantly more severe pouchitis (*p* = 0.00001).

**Conclusions:**

Neoplasia of the J-pouch is rare. Patients with neoplasia in the original specimen are more susceptible to develop neoplasia in the J-pouch. Precise follow-up in patients with neoplasia lesions in the original specimen should be recommended. Moreover, in patients with risk factors, the exact surveillance pouch endoscopy should be recommended.

## Introduction

Restorative proctocolectomy (RPC) with ileal pouch-anal anastomosis (IPAA) is a current gold standard procedure for ulcerative colitis (UC) patients who required surgical treatment.

Ulcerative colitis is a well-known risk factor for colorectal cancer (CRC). The presence of dysplasia as well as adenocarcinoma of the large bowel mucosa constitute the indications for surgical management [1]. It was also proven that the presence of CRC in the original specimen increases the risk of ileal pouch dysplasia [2, 3].

In general, the prevalence of dysplasia and adenocarcinoma in the ileal pouch is low. The cumulative incidence of ileal pouch neoplasia (both dysplasia and cancer) at 10 and 20 years in UC patients following RPC was 1.3 and 4.2 %, respectively [3]. Comparable results were presented by Derikx et al. showing the cumulative incidence of pouch neoplasia at 10 and 20 years after pouch creation to be 2.0 and 6.9 %, respectively [1]. However, the incidence of ileal pouch neoplasia is significantly higher in the group of patients with colorectal neoplasia in the original specimen [1].

Although many potential risk factors have been investigated so far, only some have been identified as a significant risk factor for ileal pouch neoplasia. Pouchitis, prior colorectal cancer, time of UC duration before RPC, concurrent primary sclerosing cholangitis (PSC), and type of IPAA (presence of mucosectomy) were the most common indicated as reported risk factors [3–7].

The first case report of pouch dysplasia was reported in 1991 [8]. Since then, there were plenty of publications regarding dysplasia and adenocarcinoma of the ileal pouch following RPC. In 2007, Scarpa et al. reported 22 cases of adenocarcinoma in pouch diagnosed worldwide, whereas 4 years later, Liu et al. presented 42 cases [7, 9]. It is believed that the longer follow-up in the group of patients after RPC is achieved, the more studies regarding ileal pouch neoplasia will be presented.

In the recent study, we proved that pouchitis may increase the risk of J-pouch dysplasia following RPC for UC patients [4]. The main goals of the present study were to assess the prevalence of neoplasia of the ileal pouch in UC patients who underwent RPC and analyze the potential risk factors for pouch neoplasia. We also investigated if there was any correlation with pouch neoplasia in patients with prior neoplasia in the original specimen of large bowel.

## Material and methods

The study was approved by the institutional bioethics committee at Poznan University of Medical Sciences.

Retrospectively, we analyzed a group of 298 patients who underwent RPC for UC in our institution between 1985 and 2009. Exclusion criteria were patients diagnosed with Crohn’s disease based on the final histopathological examination. Finally, 276 patients were included into the study. Data was collected based on the available medical records. Patients’ records were analyzed for sex, age, duration of UC before RPC, duration from pouch creation to pouch dysplasia, technique of IPAA anastomosis, presence of pouchitis, and pouchitis disease activity index (PDAI) score as well as the concurrent PSC.

RPC was performed in a standard manner. In our institution, only the J type of pouch configuration was performed. The first 27 procedures were performed with hand-sewn IPAA. In remaining patients, stapled IPAA was carried out. Depending on variety of factors (comorbidities, steroid regimen, urgent surgery, and others), two- or three-stage RPC was performed (Table 1).

**Table 1:** Characteristics of surgical management in UC patients qualified for RPC

Type of surgery	Elective	Urgent	Total
Two-stage surgery	178	28	206
Three-stage surgery	6	64	70
Total	184	92	276

Retrospectively, histopathological examination of the large bowel in all patients was evaluated. Routinely, the original specimen was prepared in the standard manner (intestine contents were flushed out and specimen was secured with 10 % buffered formalin) and sent for histopathological examination. Analysis was carried out regarding no neoplasia, CRC, or dysplasia (low-grade dysplasia (LGD) or high-grade dysplasia (HGD)) of mucosal large bowel.

Clinical examination and endoscopy of the pouch with biopsy performed yearly were routinely practiced as an institutional standard follow-up in UC patients following RPC. Pouch endoscopy was performed usually in the Pouch Outpatient Clinic with the usage of a rectoscope of various sizes (8 or 15 mm in diameter) depending on the IPAA diameter established during per rectum examination. It was performed by one of four surgeons who were well-experienced in endoscopy. Routinely, multiple biopsies were taken from (1) the body of the pouch and (2) IPAA. Additional biopsy was taken from each suspected mucosa within the ileal pouch. Routinely, we did not take biopsy of the anal transition zone (ATZ) unless there were any macroscopic changes. Histopathological examination was performed by a gastrointestinal pathologist and approved by a second pathologist.

We analyzed and performed statistical examinations of 846 biopsies performed from 2004 to 2009. Most of the examinations were performed in our center, and we included data regarding 24 endoscopic examinations from other outside centers.

Pouchitis was diagnosed based on PDAI when at least 7 points was achieved. Chronic pouchitis was diagnosed in patients in whom at least three episodes of pouchitis were diagnosed within 12 consecutive months and at least in the course of one episode pouchitis was confirmed during an endoscopic examination with biopsy [10].

The analyzed results were presented as an average ± standard deviation. As part of the statistical examination, patients were divided into two groups: patients with pouch neoplasia (*n* = 9) and patients without diagnosed foci of neoplasia in the pouch (267 patients). Chi-square test (Pearson’s test) was performed to confirm the correlations between certain characteristics such as the presence of pouch neoplasia and dysplasia or neoplasia in the large intestine before RPC, duration of UC before RPC, and intensity of PDAI. Statistically significant results were those with a *p* value <0.05. All the statistical analyses were performed using Statistica 10 (StatSoft, Inc., Tulsa, USA).

## Results

The study group comprised of 127 women (46.1 %) and 149 men (53.9 %). The mean age at the time of surgery was 43.2 years (SD 11.9). The mean time of UC duration before RPC was 11.5 years (SD 6.1; range 1–33 years), whereas the mean time of follow-up was 9.8 years (SD 6.1; range 1–23 years).

Based on the histopathological examinations of the original specimen of the large bowel, 56 patients with low-grade dysplasia and 25 patients with high-grade dysplasia were diagnosed. In five (*n* = 5) patients, adenocarcinoma of the large bowel was revealed. In the remaining samples (*n* = 190) of the original specimen of the large bowel, there was neither dysplasia nor adenocarcinoma.

**Fig. 1:**
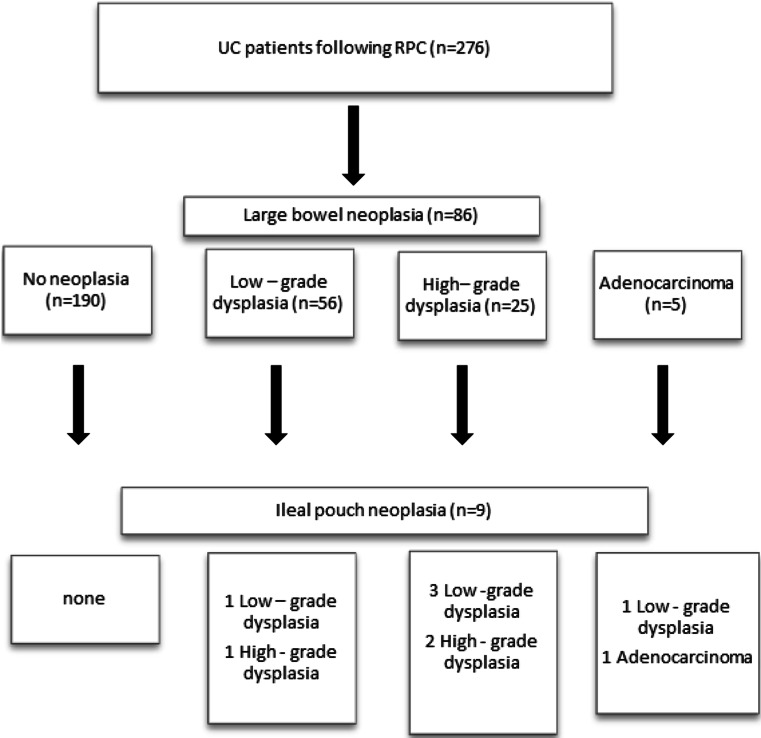
Flowchart of ileal pouch neoplasia patients with positive neoplasia lesions in the original specimen of the large bowel

Based on the available medical records, we analyzed the results of biopsies taken during routine J-pouch endoscopy. Patients’ characteristics with dysplasia and adenocarcinoma of the ileal pouch are displayed in Table 2. In five patients (*n* = 5), LGD was revealed; in three patients (*n* = 3), HGD was diagnosed; whereas in one patient (*n* = 1), adenocarcinoma was confirmed. The analyzed group of patients with J-pouch neoplasia consisted of five males (*n* = 5) and four females (*n* = 4). All reported patients with J-pouch neoplasia were positive for neoplasia in the original specimen of the large bowel (Fig. 1). The prevalence of both dysplasia and colorectal cancer in the original specimen had a significant influence on J-pouch neoplasia (*p* = 0.00001). Patients diagnosed with either pouch adenocarcinoma (*n* = 1) or HGD (*n* = 3) were qualified for pouch excision. In two patients with LGD, pouch excision was performed as well: one patient with concomitant severe pouchitis refractory to conservative treatment and in the second one because of concomitant pouch dysfunction. Remaining patients were qualified for endoscopic excision without any further repercussion. Mostly in the group of patients treated with an endoscopic approach, polypoid lesions were diagnosed. All patients diagnosed with neoplasia and treated either surgically or endoscopically required close follow-up (every 3–6 months).

**Table 2:** Patients’ characteristics with dysplasia and adenocarcinoma of the ileal pouch

Patient no.	Type of pouch neoplasia	Topographic localization of the neoplasia	Age at the time of RPC	Sex	Duration of UC before RPC (years)	Dysplasia/CRC in the original specimen	Type of IPAA	Duration from RPC to pouch dysplasia (years)	PSC	Pouchitis	PDAI
1	LGD	Pouch body	39	M	19	HGD	Stapled	7	No	Yes	9
2	LGD	NA	47	F	28	HGD	Stapled	12	No	Yes	10
3	HGD	Pouch body	40	M	19	HGD	Stapled	14	No	Yes	10
4	LGD	Pouch body	46	M	28	Ca	Stapled	18	NA	Yes	10
5	LGD	Pouch body	36	F	20	LGD	Stapled	13	No	No	6
6	Ca	Pouch body	38	F	21	Ca	Hand-sewn	20	NA	Yes	11
7	HGD	Pouch body	39	M	21	LGD	Stapled	16	No	Yes	9
8	HGD	NA	42	F	20	HGD	Hand-sewn	20	NA	Yes	10
9	LGD	Pouch body	41	M	20	HGD	Hand-sewn	18	NA	Yes	9
Total	LGD	Pouch body 4, NA 1	41.8 ± 4.7	3:2	23 ± 4.6	LGD 1, HGD 3, Ca 1	Stapled 4, hand-sewn 1	13.6 ± 4.6	No 3, NA 1	Yes 4, no 1	8.8 ± 1.6
Total	HGD	Pouch body 2, NA 1	40.3 ± 1.5	2:1	20 ± 1	LGD 1, HGD 2	Stapled 2, hand-sewn 1	17.3 ± 3.1	No 2, NA 1	Yes 3	9.7 ± 0.6
Total	Ca	Pouch body	38	0:1	21	Ca 1	Hand-sewn	20	NA 1	Yes 1	11

*LGD* low-grade dysplasia, *HGD* high-grade dysplasia, *Ca* adenocarcinoma, *PSC* primary sclerosing cholangitis, *RPC* restorative proctocolectomy, *UC* ulcerative colitis, *PDAI* pouchitis disease activity index, *IPAA* ileal pouch-anal anastomosis, *NA* not available

Duration of ulcerative colitis before RPC was revealed as a significant risk factor for pouch neoplasia (*p* = 0.01). The mean duration of UC before RPC in the group of ileal pouch neoplasia was 23 (SD 4.6), 20 (SD 1), and 21 years, respectively, for LGD, HGD, and adenocarcinoma.

The mean age at the time of pouch construction in the group of patients diagnosed with neoplasia was 40.9 (SD 3.6; range 36–47). There was no significant difference in the mean age between groups with LGD, HGD, and adenocarcinoma: 41.8 (SD 4.7), 40.3 (SD 1.5), and 38 years, respectively.

Patients with pouch neoplasia developed significantly more severe pouchitis (expressed in PDAI scale) than patients without neoplastic changes of ileal pouch mucosa (*p* = 0.00001). In the group of pouch neoplasia, the mean PDAI score was 8.8 (SD 1.6), 9.7 (SD 0.6), and 11 for patients with ileal pouch LGD, HGD, and adenocarcinoma, respectively.

Duration from pouch creation to pouch neoplasia was significantly longer in patients with ileal pouch neoplasia than in patients without pouch neoplasia (*p* = 0.0003). The more severe pouch neoplasia (LGD, HGD, and adenocarcinoma) was revealed, the longer the time following RPC was assessed. Neoplasia was revealed 13.6 (SD 4.6), 17.3 (SD 3.1), and 20 years after RPC for patients with ileal pouch LGD, HGD, and adenocarcinoma, respectively.

## Discussion

It was 1978 when Parks and Nicholls proposed RPC as a new surgical technique to keep the gastrointestinal tract continued in UC patients who required colectomy [11]. Up to date, a total of 42 adenocarcinomas of the ileal pouch have been reported [7]. Based on the review of literature regarding the incidence of pouch cancer, the topographic localization of the neoplasia was as follows: ATZ in 27 cases, pouch body in 8, both ATZ and pouch body in 2, afferent limb in 1, and remaining 4 locations were indefinite [7]. Surprisingly, based on our study, in all except two patients (data was not available) did we find pouch body as the most common topographic localization of the neoplasia following RPC.

Introduction of the stapled technique for IPAA allowed for lower perioperative complication rates as well as functional disorders of the neo-rectum resulting in anal canal sensations or sphincter disturbances [12]. However, it has raised the controversy whether leaving the mucosa of the ATZ may predispose to pouch neoplasia. According to current studies, 24 of 42 patients (57.1 %) developed pouch adenocarcinoma following hand-sewn IPAA [7]. The possible explanation of the fact is the remnant of a large-bowel mucosal islet following the mucosectomy [4]. The firm conclusion regarding the influence of the IPAA technique on ileal pouch neoplasia should not be drawn based on this study. Only the first 27 patients were operated with hand-sewn anastomosis, and in the majority of cases (*n* = 6/9), stapled IPAA was performed. However, in three patients with hand-sewn IPAA (and mucosectomy), pouch neoplasia developed.

It was proven that secondary bile acids may contribute to the increased risk of neoplasia in patients with UC and concomitant PSC [13]. Stahlberg et al. presented the relationship between the incidence of mucosal atrophy of the ileal pouch and concomitant PSC [14]. However, based on the data from Cleveland Clinic, none of the patients diagnosed with pouch adenocarcinoma suffered from PSC [2]. Because of some missing data regarding concurrent PSC in the pouch neoplasia group, we did not analyze this factor; thus, the exact conclusion should not be drawn.

Pouchitis is the most common complication of the pouch in UC patients following RPC [15]. The prevalence of pouchitis is estimated to be up to 50 % in patients after RPC for UC [16]. Chronic pouch inflammation was stated to be a risk factor for pouch neoplasia [4, 17]. It was suggested that the chronic pouchitis may predispose to atrophy of the mucosa resulting in possible dysplasia [17]. Moreover, patients who developed type C villous atrophy were more susceptible to malignancy than type A or B [18]. In the latest large studies presented by Kariv et al. and Derikx et al. based on 3203 and 1200 patients, respectively, pouchitis was not found to be an independent risk factor for neoplasia [1, 2]. However, chronic pouchitis was revealed in 3 of 11 patients (27.3 %) diagnosed with pouch adenocarcinoma [2]. Based on our histopathological examination, pouchitis was found to be a risk factor predisposing for neoplasia of the pouch. In all except one patient (LGD of the ileal pouch), the histopathological examination revealed inflammation of the pouch. The mean PDAI in the group of patients without neoplasia was significantly lower than in groups with neoplasia of the pouch.

Both dysplasia and colorectal cancer in the original specimen of the large bowel at the time of RPC were risk factors for developing neoplasia of the ileal pouch [3, 19]. Although the underlying pathology of molecular defects of DNA repair mechanisms in UC patients has been proposed, the exact pattern of the disturbances is still unknown [20, 21]. Analysis of 42 patients with pouch adenocarcinoma revealed that 24 of them (57 %) had been diagnosed with colorectal cancer before or at the time of pouch creation [7]. In the present study, in all patients who developed either pouch dysplasia or adenocarcinoma, the original specimen was positive for neoplasia.

Branco et al. found that the duration of UC before RPC in the group of patients with pouch cancer was longer than those without neoplasia (20.3 vs 11.2 years) [22]. These results are consistent with the ones presented in our study. The mean duration of IBD before RPC was significantly longer in the groups with pouch neoplasia than in patients without pouch neoplasia (20.2 vs 11.2 years).

Pouch endoscopy is a current gold standard investigation for follow-up in patients following RPC. However, the success rate in detecting dysplastic lesions of the pouch is still low. In the analyzed data of 42 patients with diagnosed adenocarcinoma of the pouch, in 23 of them, any neoplastic lesions were found before the final diagnosis of adenocarcinoma was revealed [7]. According to our results, there is a significant correlation between the presence of dysplastic changes and CRC in the original specimen of the large bowel and neoplastic lesions in the pouch. Further studies and observations are necessary to evaluate the benefits of such protocol.

The natural history of pouch neoplasia is still a topic that has not been fully examined yet. Despite the fact that potential risk factors for the development of pouch neoplasia have been defined, the precise protocol regarding the care of patients after RPC is still a topic of discussion. It has been established that there is a low incidence of pouch neoplasia. Although, some authors questioned the necessity of performing pouch endoscopy as surveillance, in our opinion, such surveillance should be recommended [23, 24]. Regular pouch endoscopy surveillance, especially among high-risk patients (prior colorectal cancer, pouchitis), seems to be a necessary element in the early diagnosis of pouch neoplasia. Moreover, in patients with a long duration of UC before RPC as well as in those with long-term follow-up after RPC, the exact surveillance pouch endoscopy should be recommended.

Based on the analyzed results in this study, it is still a question whether pouch endoscopy surveillance in the group of patients with prior colorectal neoplasia should be performed more often. The significant correlation between prior colorectal neoplasia and pouch neoplasia allows to think that the correlation is strong and associated with a high risk of pouch neoplasia.

We would like to emphasize the necessity for prophylaxis for chronic pouchitis which is a trigger for pouch neoplasia. All patients in the group with pouch neoplasia except one had a high correlation between the presence of pouchitis and the presence of pouch neoplasia, strongly suggesting initiation of a long-term prophylaxis and treatment for chronic pouchitis [25].

It should be noted that the final pathological diagnosis regarding hyperplastic and reactive adaptive changes as well as pouchitis and low-grade dysplasia may be easily confused. Thus, it is recommended to assess the histopathological specimen by at least two pathologists [26].

There are some limitations of the study. Routinely, we did not perform multifocal biopsies of the ATZ unless any suspected macroscopic lesions were revealed. As a standard in our Institution, we routinely took samples from the body of the pouch and IPAA. However, based on the study presented by Thompson-Fawcett et al., multifocal biopsy was not found as necessary in patients following RPC and UC within a duration of 10 years [27]. Moreover, it was proven in pooled published cases that the mean interval from RPC to cancer within the ATZ was 23 years whereas in the pouch body 11 years [7]. Based on our experience, we are in line with the statements that the accurate grading of J-pouch neoplasia is difficult. However, taking multifocal biopsies and evaluating samples by two independent pathologists, thus, we believe to have the accurate results optimized. From a practical point of view, it is important to analyze if there is any correlation between the prevalence of J-pouch neoplasia in groups of patients qualified for RPC because of neoplasia of the large bowel versus refractory ulcerative colitis what will be the subject of further analysis.
